# Seizures provoked by over-the-counter cough and cold medications in an elderly patient: a case report

**DOI:** 10.1186/s13256-023-03912-2

**Published:** 2023-04-29

**Authors:** Mohammed Kays Alattiya, Anwar I. Joudeh, Riyadh Ali Hammamy

**Affiliations:** Department of Internal Medicine, Al-Khor Hospital, Hamad Medical Corporation, 3050 Doha, Qatar

**Keywords:** Caffeine, Elderly, Provoked seizure, Pseudoephedrine

## Abstract

**Background:**

Seizures are common neurological emergencies in the elderly that are frequently provoked. Geriatrics have higher rates of neurological disorders and other comorbidities that could affect seizure threshold.

**Case presentation:**

An 83-year-old male Arabic patient presented to the emergency department with an acute confusional state and urinary incontinence followed by a witnessed tonic–clonic seizure in the hospital. Thorough investigations and imaging were positive only for nonspecific magnetic resonant imaging findings with a negative electroencephalogram. The patient was diagnosed with provoked seizure due to over-the-counter cold medications that included pseudoephedrine and caffeine. He was not prescribed antiepileptic medications at discharge and did not develop subsequent seizures up to this date.

**Conclusion:**

Over-the-counter cough and cold medications with sympathomimetic ingredients can be associated with provoked seizures in the elderly. Physicians should be aware of the potentially serious adverse events associated with commonly used nonprescription sympathomimetics such as pseudoephedrine and caffeine in elderly patients.

## Introduction

The diagnostic approach and management of the first attack of seizure in the elderly are often complicated by the ambiguity of seizure activity symptoms and the high rate of provoked and unprovoked seizures in this age group [1]. Earlier evidence suggested that epilepsy in older adults is associated with a faster decline in cognitive function as well as a higher 5-year mortality rate [2, 3]. Differentiating provoked from unprovoked seizures is essential to prevent further attacks and to avoid the unnecessary use of antiepileptic medications.

Drugs are among the most common causes of provoked seizures in the senile population. This could be attributed to many factors including impaired renal and/or hepatic functions, polypharmacy, and concomitant neurological diseases [4]. Multiple types of medications were found to be associated with acute symptomatic seizure in the elderly, including antimicrobials, anticancer therapy, opioid analgesics, and psychiatric medications among others [5]. Notably, several over-the-counter medications such as pseudoephedrine and caffeine work as central nervous system (CNS) stimulants and might lower the seizure threshold in the elderly [4]. In this case report, we discuss a case of an older adult who presented with a new-onset seizure after receiving a few tablets of over-the-counter cold medications.

## Case summary

An 83-year-old male Arabic patient presented to the emergency department in a public hospital in Qatar with a transient history of altered level of consciousness associated with urinary incontinence. Earlier that day, the patient was suffering from upper respiratory tract infection symptoms including a runny nose and cough for which he received one tablet of loratadine/pseudoephedrine 10 mg/240 mg and two tablets of paracetamol/caffeine 500 mg/65 mg. Later, his family found him snoring loudly in his bed with the bed sheets wet. When they tried to arouse him, he was confused and dizzy and fell from the bed, but he did not have head or body part trauma. While the patient was waiting in the emergency department, he had a witnessed attack of tonic–clonic seizure for 2 minutes. There was no history of fever, recent travel, or contact with sick patients, and his background medical history was remarkable only for type 2 diabetes mellitus and hypertension for the past 20 years. His regular medications included linagliptin, rosuvastatin, metformin, and lisinopril. The patient did not smoke cigarettes or drink alcohol before.

On examination, he had normal vital signs but was pale. He was confused about person, place, and time. However, there was no other focal neurological deficit or sign of meningeal irritation, and the rest of the physical examination was unremarkable. Initial investigations showed mild anemia [hemoglobin (Hb) 10.3 g/dL], a normal sodium level (Na 134 mEq/L), serum glucose of 8.3 mmol/L, and normal white blood cell (WBC) count and inflammatory markers (Table 1). Diagnostic studies including electrocardiogram and brain computed tomography (CT) were reported as normal. The patient received 1 g of levetiracetam after the witnessed attack in the emergency department, but no further doses were administered, and he was admitted to the hospital for 1 day for observation and further workup. However, his hospital course was uneventful.

**Table 1:** Laboratory investigation summary

Laboratory test	Value	Normal range
Hb	10.3 g/dl	13–17 g/dl
MCV	87.4 fL	83–101 fL
RDW	13.1%	11.6–14.5%
WBC	6.1 × 10^3^/μL	4–10 × 10^3^/μL
Platelet	183	150–400 × 10^3^/μL
Urea	8.7 mmol/L	2.5–7.8 mmol/L
Creatinine	109 μmol/L	62–106 μmol/L
Sodium	134 mmol/L	133–146 mmol/L
Potassium	3.5 mmol/L	3.5–5.3 mmol/L
Bicarbonate	21 mmol/L	22–32 mmol/L
Calcium	2.33 mmol/L	2.2–2.7 mmol/L
Phosphorus	0.72 mmol/L	
Chloride	99 mmol/L	
Glucose	8.3 mmol/L	
pH	7.36	
Lactic acid	1.5	
CRP	< 2 mg/L	0–5 mg/L
PT	10.9 seconds	9.7–11.8 seconds
PTT	25.4 seconds	24.6–31.2 seconds
HA1c	7.5%	
Urine leukocytes	Negative	Negative
Urine nitrates	Negative	Negative
Urine ketones	Negative	Negative
Urine glucose	Negative	Negative

*Hb* Hemoglobin; *MCV* Mean corpuscular volume; *RDW* Red Cell Distribution Width; *WBC* White Blood Count; *CRP* C-Reactive Protein; *PT* Prothrobin Time; *PTT* Partial thromboplastin time; *HA1c* Hemoglobin A1c

Brain magnetic resonance imaging (MRI) was done and showed bilateral small white-matter hyperintensities on T2 suggestive of chronic small vessel ischemic changes, while the electroencephalogram (EEG) did not show focal or generalized epileptiform discharges even with hyperventilation or intermittent photic stimulation.

At this stage, the most probable diagnosis of this patient was a drug-induced seizure. He was discharged without starting antiepileptic medications, but he was provided with seizure-specific education and to be followed in the clinic.

## Discussion

We presented a case of an elderly patient with a first-attack seizure provoked by a few tablets of over-the-counter medications for a runny nose. Inpatient investigations were unremarkable, and the patient was discharged without antiepileptic medications. No other seizure had been reported to date (around 6 months post attack).

As evident in this case, seizure activity in the elderly is often ambiguous and difficult to recognize. Initially, the patient had confusion, urinary incontinence, and falling, which are nonspecific symptoms that are common in the senile population and could be attributed to the medications he received earlier that day. The later occurrence of witnessed tonic–clonic seizure in the emergency department helped to establish the diagnosis of seizure, although motor manifestations of seizure are uncommon in the elderly [6]. According to Rowan *et al.*, the most common form of seizure in the geriatric population was focal seizure with impairment of awareness [7]. Moreover, auras, if occurring, are usually nonspecific in the form of dizziness or confusion, and post-ictal confusion could unusually be prolonged for days [8]. Of note, generalized-onset seizures are infrequent in the elderly and might indicate diffuse cerebral pathology due to aging or concomitant neurodegenerative diseases, or secondary to environmental exposure as proposed in this case [8]. Common causes for provoked and unprovoked seizures in the elderly are summarized in Table 2 (source reference [9]).

**Table 2:** Common causes for provoked and unprovoked seizures in the elderly [9]

Causes of provoked seizures	Relative proportion (%)
Acute stroke	50%
Metabolic derangement	6–30%
Secondary to medications/alcohol	10%
Others (intracranial hemorrhage, intracranial infection, and so on)	5–20%
Causes of unprovoked seizures	Relative proportion (%)
Cerebrovascular disease	30–50%
Dementia	9–17%
Others (previous head injury or infection, brain tumor, and so on)	5–15%
Unknown	30–50%

Reference: Shih T. Seizures and epilepsy in older adults: Etiology, clinical presentation, and diagnosis [Internet]. Schachter S, Schmader K, Dashe J, editors. UpToDate. 2021 [cited 2023Mar7]. Available from: https://www.uptodate.com/ [9]

Given the atypical presentation of seizures in the elderly, other neurological emergencies with similar semiology should be promptly ruled out. Cerebrovascular events, intracerebral hemorrhage, and delirium may also present similarly in senile patients where timely diagnosis and management are vital to reduce potentially associated mortality and morbidity. In addition to the initial laboratory and clinical evaluation to exclude metabolic derangement, arrhythmias, and orthostatic hypotension, neuroimaging studies play an essential role in excluding differential diagnoses and identifying focal lesions that might trigger seizure activity [10]. In this case, an emergency noncontrast CT scan did not reveal significant intracranial pathology, and a subsequent brain MRI showed few nonspecific white-matter lesions suggestive of chronic small vessel ischemic changes. According to Borja *et al.*, abnormal findings on head CT scans for adults with first-time seizures vary between 3% and 40%. However, CT scans are still required for elderly patients in the emergency department as they rapidly exclude bleeding, intracranial masses, and large infarcts. Nevertheless, head MRI provides a better assessment of brain parenchyma including focal lesions, infarcts, tumors, microhemorrhages, and gliosis [10].

Neurological and cardiovascular adverse events including death have been repeatedly reported in the pediatric literature with therapeutic and supratherapeutic doses of over-the-counter cough and cold medications [11]. However, few cases were reported in adults. İsmailoğulları *et al.* described an attack of nonconvulsive status epilepticus in a 31-year-old apparently healthy woman after using two types of cough and cold medications containing pseudoephedrine, dextromethorphan, paracetamol, and chlorpheniramine for 10 days [12]. Unlike our case, this patient had a single episode of complex partial seizure at the age of 8 years but she was not receiving any pharmacological treatment for it. Beside this report, ephedra-containing dietary supplements were also reported to have a temporal association with seizure in adults with no prior history of seizure. However, in that case series of adverse events, ephedra was commonly used in combination with other stimulants [13].

Caffeine, which was also used in this case, is considered a CNS stimulant with a complex relationship with seizure. According to a systematic review and qualitative analysis of 105 studies, caffeine can trigger seizures in patients with and without a history of epilepsy, and even a single high dose of caffeine could lower the threshold for seizure. Moreover, caffeine interferes with antiepileptic drugs, especially topiramate, which might lower their efficacy. On the other hand, chronic low doses of caffeine seem to protect from seizures in animal studies [14]. On this basis, inquiry about dietary and over-the-counter use of caffeine should be included in the assessment of patients presenting with new-onset seizures or changes in the pattern of seizure control.

Considering the increasing number of geriatric populations and challenging physical examination, the role of detailed history and drug exposure for senile patients with seizures could not be overrated. Patients and physicians should be aware of the neurophysiological adverse effects of nonprescription sympathomimetic medications including pseudoephedrine and caffeine. Further studies are needed to clarify the relationship between certain over-the-counter cough and cold medications and seizure precipitation. Such studies will also help in risk stratification prediction and the development of regulatory roles that guide the use of these commonly used drugs.

## Conclusion

This report described a case of provoked generalized seizure in an older individual with no prior history of seizure after exposure to a small dose of pseudoephedrine combined with caffeine. Initial evaluation of elderly patients presenting with new-onset seizure should include detailed history of prescription and nonprescription drug use. The use of certain over-the-counter sympathomimetic medications could pose serious health risks to elderly patients; hence, they should be used with caution.

## Data Availability

Data can be obtained from the corresponding author upon request.

## Abbreviations

CT: Computed tomography
EEG: Electroencephalogram
MRI: Magnetic resonance imaging
